# From glossy to glaucous: How *TaMYB96-2D* controls wax deposition and drought resilience

**DOI:** 10.1093/plphys/kiaf675

**Published:** 2025-12-24

**Authors:** Ritu Singh, Erin Cullen

**Affiliations:** Assistant Features Editor, Plant Physiology, American Society of Plant Biologists; Department of Plant Science, University of California, Davis, CA 95616, United States; Assistant Features Editor, Plant Physiology, American Society of Plant Biologists

The plant cuticle is a hydrophobic barrier that covers aerial plant organs and provides protection against biotic and abiotic stresses, including pathogens, herbivores, drought, and excess light (González-Valenzuela et al. 2023). The cuticle limits water loss and restricts access to underlying epidermal cells. Structurally, it consists of a cutin polyester matrix overlaid and impregnated with cuticular waxes derived from very long chain–fatty acids. These wax components largely determine cuticle permeability and surface properties, directly linking wax composition to cuticle function. Cuticle composition varies among plant species, organs, and developmental stages, reflecting the need to adapt barrier properties to distinct environmental and developmental contexts (Ingram and Nawrath 2017).

Although several enzymes involved in cuticular wax biosynthetic enzymes have been characterized, the transcriptional regulators that coordinate this complex wax production to shape cuticle structure and function remains elusive. In a recent issue of *Plant Physiology*, Wang et al. (2025) identified TaMYB96-2D, a wheat R2R3 MYB transcription factor, as a master regulator of wax production and glaucousness (a blue-gray waxy coating on wheat organs that reflects light).

The authors identified 3 *MYB* genes, *TaMYB96-2A*, *TaMYB96-2B*, and*TaMYB96-2D*, located on wheat subgenome 2A, 2B, and 2D. Although similar, *TaMYB96-2D* shows the highest identity to Arabidopsis *MYB94* and *MYB96*, which are well known regulators of wax biosynthesis (Lee et al. 2016), making it the most promising candidate for functional characterization. To identify its biological roles, the authors generated a CRISPR-mediated triple knockout that removed all 3 *TaMYB96* genes and also created transgenic lines overexpressing *TaMYB96-2D*. Plants overexpressing *TaMYB96-2D* displayed enhanced glaucousness and visibly thicker wax deposits across leaves, sheaths, peduncles, and glumes. In contrast, knockout plants were glossy, with non-glaucous surfaces lacking the typical blue-gray bloom. Scanning electron microscopy imaging further revealed that knockout plants showed disrupted organization of tubule- and platelet-shaped wax crystals that scatter light and create glaucousness.

The authors further quantified wax amounts and composition across aerial tissues using gas chromatography-mass spectrometry and gas chromatography-flame ionization detection. Knockout plants showed major reductions across all wax classes, with diketones showing the most dramatic decrease, especially in reproductive organs such as glumes. Diketones are the dominant wax constituents that form the tubular crystals associated with glaucousness, which explains the nonglaucous, glossy phenotype of the knockout lines. Conversely, overexpression of *TaMYB96-2D* increased total wax loads. Further, chain length analysis showed that *TaMYB96-2D* not only regulates the amount but also the type of wax molecules produced, as knockouts displayed reductions in C20-C28 primary alcohols, C31 diketones, and C27-C33 alkanes, while overexpression lines accumulated higher levels of these long-chain molecules. These results collectively establish *TaMYB96-2D* as a broad regulator that coordinates both the quantity and quality of wheat wax.

To understand how *TaMYB96-2D* controls these pathways, the authors analyzed the expression of key wax biosynthetic genes. Knockout plants showed significantly reduced expression of key diketone biosynthetic genes (*TaDMH*, *TaDMP*, *TaDMC*), alkane biosynthetic genes (*TaCER1-1A*/*6A*), and primary alcohol biosynthetic genes (*TaFARs*). These same genes were strongly upregulated in overexpression lines, suggesting that *TaMYB96-2D* works upstream of multiple wax biosynthetic pathways. Earlier work showed that TaMYB96-2D binds to a conserved CAACCA motif in wax-related promoters (He et al. 2022). In the current study, yeast 1-hybrid assays, GUS and luciferase assays, and chromatin immunoprecipitation assay qPCR confirmed that TaMYB96-2D binds directly to the promoters of *TaDMH*, *TaDMP*, and *TaDMC* (Fig. 1). This provided strong molecular evidence that TaMYB96-2D activates the enzyme pathways that produce diketones, alkanes, and primary alcohols.

**Figure 1.**
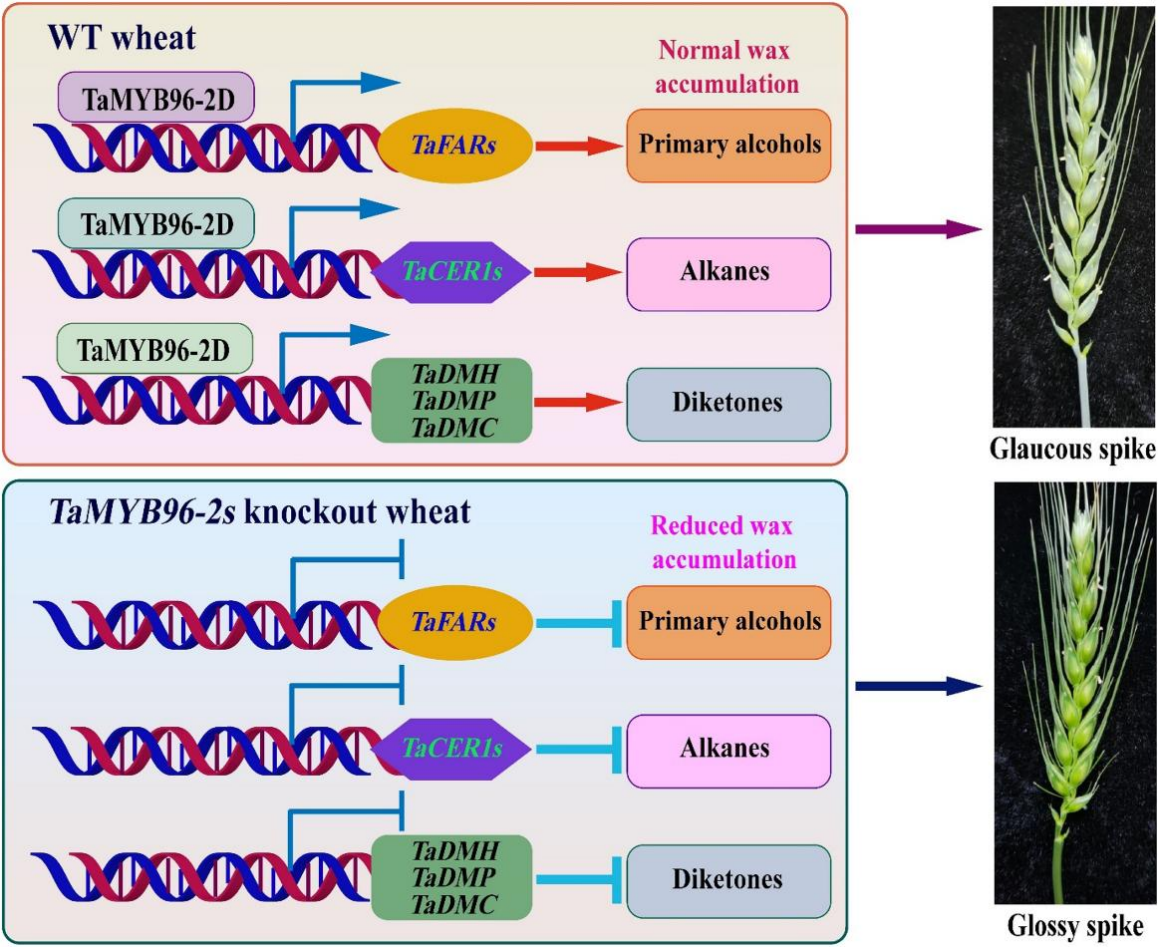
A proposed model for the role of *TaMYB96-2D* in regulating cuticular wax biosynthesis in wheat (adapted from Figure 8 from Wang et al. 2025). The transcription factor TaMYB96-2D can promote cuticular wax accumulation by direct activation of biosynthetic wax genes in the wild-type wheat plants, causing a glaucous appearance. In *TaMYB96-2s* knockout wheat, the expression of wax biosynthetic genes is inhibited, leading to a reduction in cuticular wax accumulation and thereby resulting in a glossy green appearance.

As glaucousness often correlates with abiotic stress resilience (Li et al. 2019; Jian et al. 2022), the authors tested whether *TaMYB96-2D* functions in abiotic stress adaptation. *TaMYB96-2D* expression increased strongly in response to drought, salinity, cold, heat, and abscisic acid. The gene was also induced by polyethylene glycol treatment, which mimics osmotic stress and reduces water availability to the plant. These responses indicate that *TaMYB96-2D* links stress signaling with cuticle reinforcement. Drought-stressed plants also accumulated more wax, matching the increase in *TaMYB96-2D* expression. The functional consequences were tested through cuticle permeability assays. Overexpression lines exhibited reduced cuticle permeability, slower chlorophyll leaching, and reduced water loss, whereas knockout plants showed the opposite trend and lost water rapidly. These surface-level changes are translated into whole plant responses. During water deprivation, knockouts wilted rapidly and had poor recovery, while overexpression plants maintained greener tissues and had higher survival rates. Notably, stomatal density and conductance were unchanged, confirming that the drought tolerance effects arise from cuticle-based water retention rather than stomatal adjustments.

An evolutionary analysis of 53 global wheat accessions added another layer of insight. One haplotype of *TaMYB96-2D*, named Hap I, was found in 46 of the accessions. The high frequency of this haplotype suggests that breeding has favored *TaMYB96-2D* alleles that improve wax accumulation and stress resilience. This connects molecular function with long-term selection in modern wheat.

Together, this work establishes *TaMYB96-2D* as a central transcriptional switch that links environmental signals, wax biosynthesis, and drought resilience in wheat. By activating multiple wax pathway genes, *TaMYB962D* drives the production of long chain lipids that strengthen the cuticle and help wheat maintain water balance under stress (Fig. 1). This work opens new opportunities for breeding wheat varieties with improved drought tolerance by manipulating a single upstream regulator of cuticular wax biosynthesis. Future studies should also examine whether *TaMYB962D* contributes to defense against biotic stresses, as cuticle integrity often influences pathogen entry and disease resistance.

Recent related articles in *Plant Physiology*:

Wang et al. (2025) showed that the SAGA histone acetyltransferase complex acts with RNA processing machinery to regulate cuticular wax biosynthesis in wheat, likely through control of *TaCER3* expression.

Xu et al. (2024) identified genetic loci controlling cuticular wax variation in maize and showed that *ZmKCS12* regulates wax composition, leaf traits, and drought tolerance.

## Data Availability

No data is generated in this study.
